# Altered Glutamate Receptor Ionotropic Delta Subunit 2 Expression in Stau2-Deficient Cerebellar Purkinje Cells in the Adult Brain

**DOI:** 10.3390/ijms20071797

**Published:** 2019-04-11

**Authors:** Helena F. Pernice, Rico Schieweck, Mehrnoosh Jafari, Tobias Straub, Martin Bilban, Michael A. Kiebler, Bastian Popper

**Affiliations:** 1 Biomedical Center (BMC), Department for Cell Biology & Anatomy, Medical Faculty, Ludwig-Maximilians- University, 82152 Martinsried, Germany; Franziska.Pernice@med.uni-muenchen.de (H.F.P.); Rico.Schieweck@med.uni-muenchen.de (R.S.); 2 Institute of Clinical Neuroimmunology, University Hospital and Biomedical Center, Ludwig-Maximilians-University, 81377 Munich, Germany; mehrnoosh.jafari@med.uni-muenchen.de; 3 Biomedical Center (BMC), Core Facility Bioinformatics, Medical Faculty, Ludwig-Maximilians-University, 82152 Martinsried, Germany; tstraub@bmc.med.lmu.de; 4 Department of Laboratory Medicine and Core Facility Genomics, Medical University of Vienna, 1090 Vienna, Austria; martin.bilban@meduniwien.ac.at; 5 Biomedical Center (BMC), Core Facility Animal Models, Medical Faculty, Ludwig-Maximilians-University, 82152 Martinsried, Germany

**Keywords:** staufen2, synaptogenesis, cerebellum, purkinje cells, GluD2

## Abstract

Staufen2 (Stau2) is an RNA-binding protein that is involved in dendritic spine morphogenesis and function. Several studies have recently investigated the role of Stau2 in the regulation of its neuronal target mRNAs, with particular focus on the hippocampus. Here, we provide evidence for Stau2 expression and function in cerebellar Purkinje cells. We show that Stau2 downregulation (Stau2^GT^) led to an increase of glutamate receptor ionotropic delta subunit 2 (GluD2) in Purkinje cells when animals performed physical activity by voluntary wheel running compared with the age-matched wildtype (WT) mice (C57Bl/6J). Furthermore, Stau2^GT^ mice showed lower performance in motor coordination assays but enhanced motor learning abilities than did WT mice, concomitantly with an increase in dendritic GluD2 expression. Together, our results suggest the novel role of Stau2 in Purkinje cell synaptogenesis in the mouse cerebellum.

## 1. Introduction

Research over the last few decades has shown the importance of posttranscriptional regulation mechanisms in neuronal function, particularly for synaptogenesis [1]. Key players for these processes are RNA-binding proteins (RBPs) [2,3]. By binding to their target mRNAs, RBPs control different steps of the RNA life cycle, such as splicing, RNA export and localization, translation control and degradation [1] underlining their importance for neuronal functioning. Depletion of RBPs can cause severe neuropathologies, such as mental retardation or epilepsy, as well as motor defect disorders, such as amyotrophic lateral sclerosis (ALS) [4,5,6,7]. Staufen2 (Stau2) is an RBP involved in different regulatory networks ranging from embryonic neurogenesis to synaptic transmission and morphogenesis in mature hippocampal neurons [8]. Recent studies of Stau2-deficient mice and rats have shown that Stau2 is important for learning and memory formation [9,10]. In this study, we took advantage of the previously reported Stau2 gene trap mouse model (Stau2^GT^), which shows a brain-wide Stau2 downregulation of about 40% [9]. Transcriptome-wide analysis of differentially expressed RNA in the Stau2^GT^ mouse brain revealed an upregulation of *glutamate receptor ionotropic delta subunit 2* (*Grid2)* and *cerebellin1* (*Cbln1*) mRNAs when compared with wildtype (WT) mice during extensive learning and motor activity. These mRNAs code for glutamate receptor ionotropic delta subunit 2 (GluD2) and Cbln1, two components of the cerebellar synaptic apparatus [11,12]. GluD2 and Cbln1 are believed to connect the synapses of granule cell parallel fibers and Purkinje cell dendrites. While GluD2 is expressed in the postsynaptic membrane of Purkinje cells, Cbln1 is secreted from the presynaptic compartment, binds to presynaptic neurexins and postsynaptic GluD2, and therefore connects pre-synapses deriving from parallel fibers of granule cells with post-synapses of Purkinje cells [13,14]. The consolidation of synapses through the interaction of these molecules has been shown to be the basis of synaptic transmission in Purkinje cells. Furthermore, Cbln1 and GluD2 play relevant roles in motor coordination and learning, while deficits lead to motor impairment, such as ataxia in mouse models [15,16,17]. Interestingly, naive Stau2^GT^ showed reduced motor coordination in a rotor rod assay compared with age-matched WT animals. Strikingly, mutant animals displayed enhanced motor learning skills during repeated training. These effects were concomitant with an increased GluD2 expression, suggesting improved synaptic transmission in the cerebellum. Together, our results suggest that Stau2 plays a role in motor activity-induced synaptogenesis in the mouse cerebellum.

## 2. Results

### 2.1. Adult Stau2^GT^ Mice Show an Upregulation of Cbln1 and GluD2 mRNA during Behavior Testing

**Figure 1:**
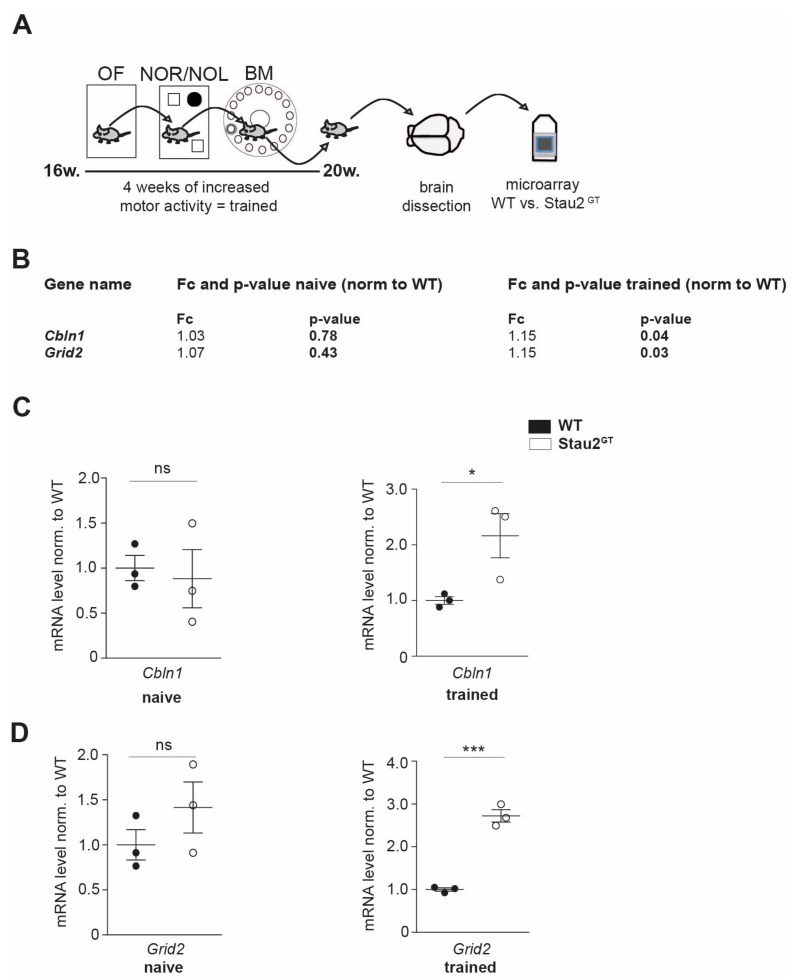
Adult Staufen2 knock-down (Stau2^GT^) mice show an upregulation of *cerebellin1* (*Cbln1*) and *glutamate receptor ionotropic delta subunit 2* (*Grid2*) mRNA during behavioral tests. (**A**) Schematic representation of the experimental approach followed by the behavioral tests. Here, 4-month-old Stau2^GT^ and wildtype (WT) mice were exposed to a battery of behavioral tests (OF = open field test, NOR/NOL = novel object recognition/location test, and BM = Barnes maze), enabling physical trainings (= trained). Subsequently, the mouse brains were dissected, and microarray analysis was performed. (**B**) Microarray analysis of brain lysates of 5-month-old Stau2^GT^ and WT mice before and after training conditions. (**C**,**D**) Quantification of *Cbln1* (C) and *Grid2* (D) mRNA levels before (naive) and after behavioral tests (= trained) measured by qRT-PCR. *N* = 3 animals (**B**–**D**). Statistics: Students t-test. Mean + SEM, * *p* < 0.05, *** *p* < 0.001; ns = not significant (**B**–**D**).

To identify genes that are Stau2-dependently affected during training, 4-month-old Stau2^GT^ mice and age-matched WT controls were exposed to a battery of behavioral tests for 4 consecutive weeks, as previously described [8] (Figure 1A). Subsequently, the mouse brains were dissected, and microarray analysis was performed. Interestingly, compared with the WT mice, we detected a significant 15% increase in the Stau2^GT^ mice for *Cbln1* and *Grid2* mRNA expression in brain lysates, which code for cerebellar proteins involved in synaptogenesis [12,18]. In naive mice, in contrast, there was no apparent changes in the expression of these mRNAs detectable (Figure 1B). To validate the upregulation of *Cbln1* and *Grid2* mRNAs, qRT-PCR was performed on Stau2^GT^ and control brain lysates. Here, mRNA levels of *Cbln1* and *Grid2* showed a significant increase in the adult Stau2^GT^ mice that underwent behavioral experimentation (Figure 1C,D).

### 2.2. Reduced Stau2 Expression in Cerebellar Purkinje Cells of Adult Stau2^GT^ Mice

**Figure 2:**
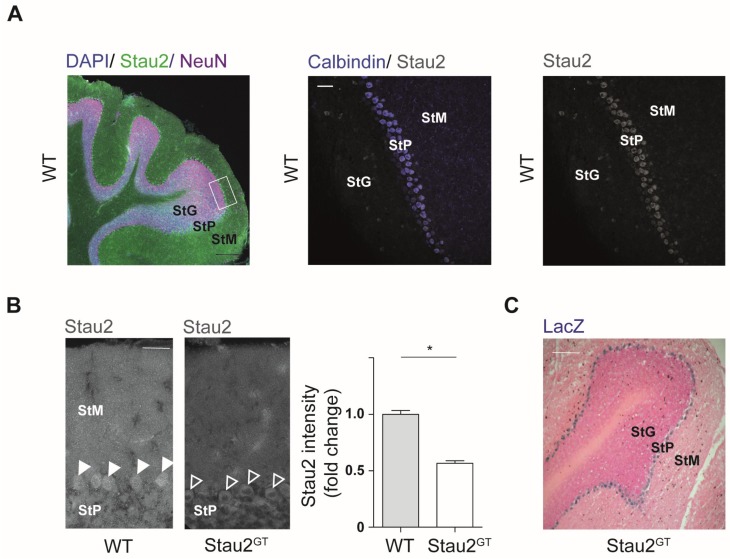
Stau2 expression in cerebellar Purkinje cells is reduced in adult Stau2^GT^ mice. (**A**) Representative confocal images of immunohistofluorescence staining for Stau2 (neuronal markers: NeuN and calbindin; nuclear marker: 4′,6-diamidino-2-phenylindole (DAPI)) on frontal sections of cerebelli taken from 5-month-old WT mice. Scale bars = 200 µm (left), 50 µm (middle, right). (**B**) Representative confocal images of immunohistofluorescence staining against Stau2 on frontal sections of the cerebellum of 5-month-old Stau2^GT^ and WT mice (left) and relative Stau2 intensity (right). Scale bar = 20 µm. (**C**) Light microscopic image of LacZ staining in a representative frontal section of Stau2^GT^ mouse cerebellum. Scale bar = 200 µm. *N* = 3 (**B**). Statistics: Students *t*-test. Mean + SEM, * *p* < 0.05. StG, stratum granulare; StP, stratum pyramidale; and StM, stratum moleculare. Arrows point to Purkinje cell bodies (**B**).

The increase in expression of the cerebellar genes *Cbln1* and *Grid2* prompted us to examine Stau2 expression in the mouse cerebellum. First, we performed immunostainings against Stau2 in frontal cerebellar sections in adult WT mice. Co-staining for calbindin was used to clearly distinguish Purkinje cells from NeuN-expressing cells. Stau2 staining was prominent in the somatic area of the Purkinje cell layer (StP) and the stratum moleculare (StM) (Figure 2A). To evaluate Stau2 downregulation in the cerebellum of transgenic mice, we performed immunostainings against Stau2 in cerebellar sections of Stau2^GT^ mice. Quantification of Stau2 protein intensity in Purkinje cells showed a 50% reduction in adult Stau2^GT^ animals compared with the controls (Figure 2B). Furthermore, staining for the β-galactosidase activity encoded by the gene trap construct in Stau2^GT^ mice [9] revealed a strong expression in Purkinje cells, indicating a knockdown preferentially in the stratum purkinjense (Figure 2C) underlining our previous results (Figure 2B).

### 2.3. Decreased Motor Coordination Abilities but Increased Motor Learning Capacity in Adult Male Stau2^GT^ Mice

**Figure 3:**
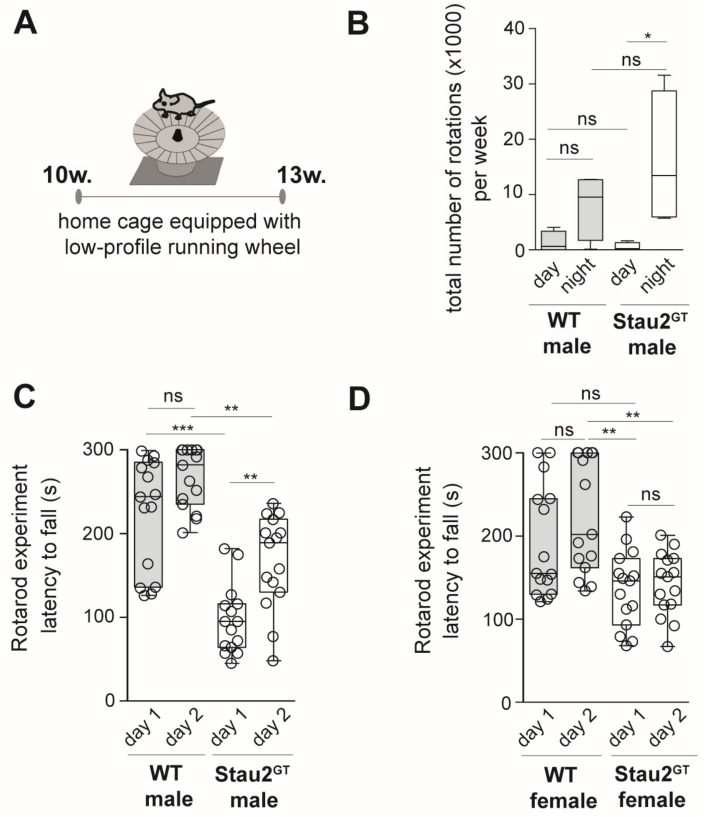
Adult male Stau2^GT^ mice show decreased motor coordination abilities but increased motor learning capacity. (**A**) Setup for voluntary wheel running experiments. Low-profile running wheels as depicted were placed into the home cages of 10-week-old Stau2^GT^ mice and age-matched WT controls for 3 weeks (w.). (**B**) Graph depicting the number of total rotations per week at night and day used by either the WT or Stau2^GT^ mice. (**C**) Quantification of rotarod running wheel testing. Here, 10-week-old male (left) or female (right) Stau2^GT^ mice, as well as age- and gender-matched controls, were set on the rotarod three times per day on two consecutive days and time until fall off was measured (the mean value per day was calculated). Mean latency to fall (in seconds) for either the WT or Stau2^GT^ male or female mice is plotted as boxplots. Individual data points represent different trials for single animals per day. *N* = 5 animals/group. Statistics: mean + SEM (B) or mean + min/max (**C**/**D**), one-way ANOVA and Bonferroni’s multiple comparison test. * *p* < 0.05, ** *p* < 0.01, *** *p* < 0.001, ns. = not significant.

The fact that Stau2 was found to be expressed in Purkinje cells and that its downregulation affected the gene expression of cerebellar proteins during behavior testing prompted us to investigate the impact of Stau2 depletion on motor activity and coordination. First, we measured the overall motor activity of adult Stau2^GT^ and WT mice exposed to a low-profile running wheel [19] during a three-week period (Figure 3A). Interestingly, the Stau2^GT^ mice showed a significantly higher tendency to use the running wheel at night (6 p.m.–6:00 a.m.) when compared with the tendency observed in the WT mice (Figure 3B), indicating that Stau2 does not affect general motor activity. To investigate the effect of Stau2 downregulation on motor coordination, we used the rotarod performance test [20] (Figure 3C). Then, 10-week-old Stau2^GT^ and WT mice were exposed to the rotarod running wheel. The duration of running until the animals fell from the wheel was measured in tests performed over two consecutive days. We observed that both Stau2^GT^ males and females showed a lower latency to fall than the age- and sex-matched controls on day 1 (Figure 3C,D). On day 2, male Stau2^GT^ mice improved significantly their test performance compared with the age-matched WT males (compare day 1 and day 2 for the Stau2^GT^ and WT animals), although the general performance was still lower in the Stau^GT^ mice (Figure 3C). Interestingly, for Stau^GT^ females, we did not observe a performance improvement during training (Figure 3D). Our expression analysis using mutant and WT brains from males (Figure 1) mirrored the effect that we observed in rotarod experiments. We therefore continued with male mice.

### 2.4. Increased Dendritic GluD2 Protein Expression in Adult Stau2^GT^ Mice after Motor Activity.

**Figure 4:**
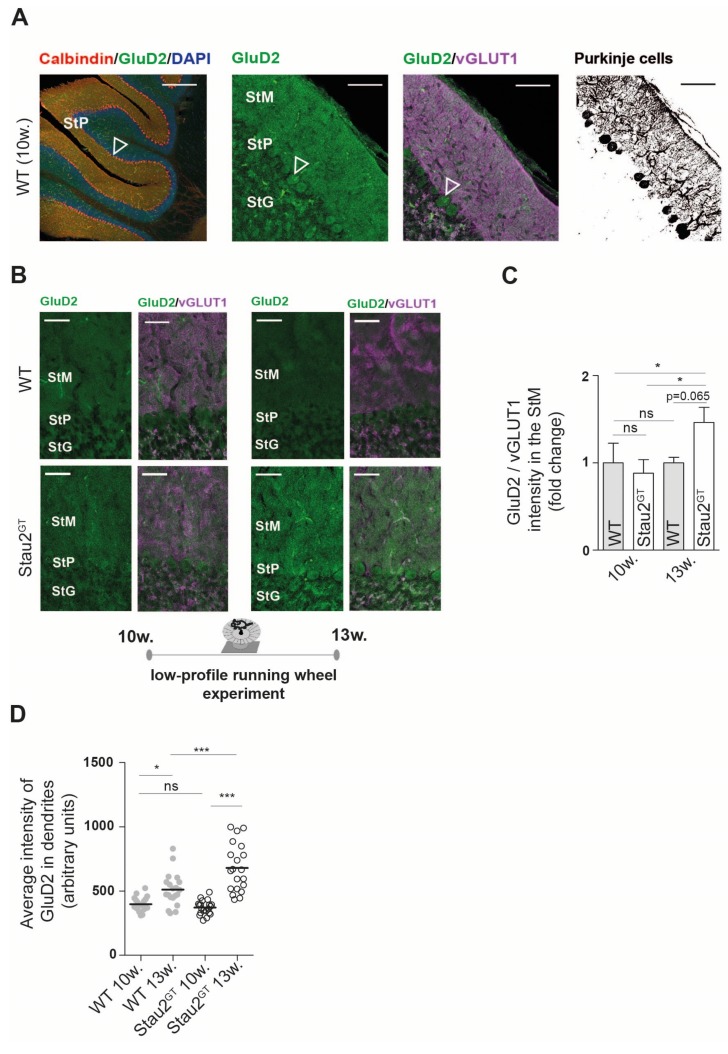
Dendritic GluD2 protein expression increases in adult Stau2^GT^ mice after motor activity. (**A**) Representative confocal images of immunofluorescent staining against GluD2, (vesicular glutamate transporter 1) vGLUT1, and calbindin (negative conversion for Purkinje cell morphology) on frontal sections of the cerebellum taken from 5-month-old WT mice. Scale bar = 200 µm (left), 100 µm (middle, right); arrow points to Purkinje cell bodies. (**B**) Representative confocal images of immunofluorescent staining against GluD2 and vGLUT1 in the Stau2^GT^ and WT mice before and after exposure to the running wheel (setup Figure 3). Scale bar = 40 µm. (**C**) Quantification of the mean staining intensity of GluD2 normalized to the mean staining intensity of vGLUT1 before (10 w. = mice at the age of 10 weeks) and after (13 w. = mice at the age of 13 weeks) 3 weeks of voluntary wheel running. *N* = 3. Statistics: Student’s t-test. Mean + SEM. (**D**) Histogram showing the average intensity of the GluD2 signal in calbindin-positive cerebellar Purkinje cell dendrites before (10 w.) and after (13 w.) running wheel exposure. The WT (filled grey circles) and Stau2^GT^ mice (empty black circles). *N* = 20 dendritic stretches in each group, from three different animals. Statistics: One-way ANOVA and Bonferroni’s multiple comparison. Mean +SEM, * *p* < 0.05, ** *p* < 0.01, *** *p* < 0.001; ns. = not significant. StM, stratum moleculare; StP, stratum pyramidale; and StG, stratum granulare.

Based on our microarray and rotarod test results and because enhanced motor activity induces synaptogenesis in the cerebellum [21], we speculated that Stau2 might play a role in this process. Therefore, we analyzed histological sections of the cerebellar cortex. We stained against GluD2 protein and vesicular glutamate transporter 1 (vGLUT1), a protein located at the presynaptic side of synapses between parallel fibers and Purkinje cells [22] (Figure 4A). Naive Stau2^GT^ mice (10 weeks old) showed weak expression of GluD2 in the StM and cell bodies of Purkinje cells. In contrast, motor activity by voluntary wheel running (3 weeks) increased GluD2 expression in the Stau2^GT^ mice by 50%, whereas no difference in GluD2 was observed in the WT animals. Furthermore, we detected elevated GluD2/vGLUT1 colocalization in the StM, suggesting an increase in the number of functional synapses in Stau2^GT^ mice after motor activity (Figure 4B,C). We next analyzed the GluD2 intensity in Purkinje cell dendrites of naive mice, as well as mice exposed to running wheels. Here, we detected higher levels of dendritically localized GluD2 protein in cerebellar Purkinje cells (Figure 4D). Naive 10-week-old WT and Stau2^GT^ mice, in contrast, showed no difference in GluD2 protein expression. Confirming previous experiments, the effect was much stronger in the Stau2^GT^ mice after motor activity than in the WT mice (Figure 4D). In summary, the Stau2^GT^ mice showed higher voluntary motor activity, a steeper motor learning curve, and a more prominent increase of dendritic GluD2 expression after motor activity.

## 3. Discussion

Stau2 is an essential RNA-binding protein involved in posttranscriptional gene regulation in the brain [8,23,24]. Its role in hippocampus-dependent learning and memory formation has been studied in Stau2-deficient rat and mouse animal models [9,10]. However, little is known about its function in other brain regions. Here, we report a potential impact of Stau2 on synaptogenesis in the mouse cerebellum. Increased physical activity during behavior testing induced increased expression of *Grid2*, *Cbln1*, and *Cbln3* mRNA in adult Stau2^GT^ mice, which act together in the formation of parallel fiber-Purkinje cell synapses [11]. Interestingly, *Grid2* was detected in an iCLIP Stau2 study suggesting that it is a target of Stau2 [24]. Furthermore, the Stau2^GT^ mice showed lower motor coordination abilities but increased motor learning capacity in the rotarod test. Of note, we did not observe a similar effect in female Stau2^GT^ mice. Because we aimed at reducing the animal number used in our experiments, leading to a number of five mice per group, a larger cohort of females might be needed to observe enhanced motor learning skills. Similar to exposure to voluntary physical exercise experiments, these repeated rotarod trainings correlated to increased GluD2 protein expression in parallel fiber dendrites of cerebellar Purkinje cells.

The importance of GluD2 as a key component of the Purkinje cell post-synapse, thereby inducing synapse formation by interacting with presynaptic neurexins through secreted Cbln1, has been reported by several groups in the last few years [11,12,13,14,25]. GluD2 knockout (KO) mouse models have demonstrated a prominent motor dyscoordination and cerebellar ataxia [16,26,27]. Rotarod tests performed by GluD2 KO mice have resulted in a lower latency to fall [27], identifying GluD2 as an essential regulator of cerebellar synaptic transmission and, eventually, of motor coordination. Consequently, it is tempting to speculate that higher expression of GluD2 in Stau2^GT^ males during training contributes to the enhanced learning of motoric skills in these mice.

A possible basis of reduced motor coordination and motor learning in GluD2 KO mice is reduced long-term depression (LTD) in the cerebellum [28,29,30]. Interestingly, reduced Stau2 expression also leads to the disruption of LTD in the hippocampus [10,31]. Reduced LTD in the cerebellum caused by Stau2 deficiency might explain the generally weaker performance of adult Stau2^GT^ mice in rotarod tests compared with WT mice. Another possible reason for the lower test performance of Stau2^GT^ mice could be their generally reduced locomotor activity in a new environment, as has recently been reported [9].

Besides GluD2, we observed increases in *Cbln1* mRNA after stimulation of motor activity through behavioral tests in Stau2^GT^ mice when compared with WT mice. We interpret this result as a feedback response to enhanced synaptogenesis in adult Stau2^GT^ mice compared with WT animals after stimulation of motor activity. Cbln proteins are secreted by cerebellar granule cells and are predominantly expressed in the cerebellum, although involvement in other brain areas, such as the frontal brain have been reported [32,33,34]. *Cbln1*-null mice exhibit a cellular and physiological phenotype mimicking that of the GluD2 KO, underlining the tight functional interplay between these two proteins [32]. In another study, cerebellar ataxic gait in *Cbln1*-null mice was found to be clinically improved through Cbln1 injections into the cerebellum [35].

In summary, our results suggest that Stau2 is involved in Purkinje cell synaptogenesis in the adult mouse cerebellum and might contribute to the regulation of motor coordination and learning. These findings therefore hold consequences for new clinical approaches. For example, GluD2 mutations have been shown in cases of congenital cerebellar ataxia in patients [17]. A better understanding of the involvement of Stau2 in GluD2-dependent synapse formation could therefore provide new therapeutic options for cerebellar ataxia and similar disorders. It will be interesting to investigate further gender-specific differences in Stau2 function. Future studies are needed to unravel the impact of Stau2 in cerebellar synaptogenesis at the molecular level and its relevance for motor coordination and activity defects in humans.

## 4. Materials and Methods

### 4.1. Generation and Housing of Stau2 Gene Trap Mice

All experiments were performed according to local animal protection laws and were approved by the district government of upper bavaria (55.2-1-54-2532-167-2013, 23 May 2014) Adult B6.129P2-Stau2^Gt(RRG396)Byg^ mice [9] and WT C57Bl/6J mice (aged 10–13 weeks and 16–20 weeks) were housed in groups of 2–5 animals with a 12-h light/12-h dark light cycle in individually ventilated cages. The mice had free excess to autoclaved water and food. 

### 4.2. Behavioral Analysis

All behavioral tests were performed under identical environmental conditions. Between individual testing phases, the animals were kept in their housing cages. First, 4-month-old male Stau2^GT^ and WT mice were exposed to a stimulation of exploratory and motor behavior for 4 consecutive weeks via different behavioral tests (open field, novel object recognition/location, Barnes maze) as described previously [9].

*Running wheel*: Low-profile running wheels (Med associates Inc, Fairfax, VT, USA) were placed in home cages of age- and gender-matched Stau2^GT^ and WT mice for a time period of 3 weeks. Motor activity was tracked by measuring total rotations of the running wheel per day. 

*Rotarod test:* The rotarod test was performed as previously described [36]. Age- and gender-matched Stau2^GT^ and WT mice were set on a rotating rod (accelerating rotations during 5-min test duration), and the latency until fall from the rod was measured in seconds. The test was performed for two consecutive days. WT male and Stau2^GT^ males, as well as WT female and Stau2^GT^ female mice, were investigated.

### 4.3. Histology

Whole animal perfusion fixation was performed as previously described [37]. Cerebellar tissue was prepared as previously described [38]. 

*Immunohistochemistry*: Brains of perfusion fixed mice were postfixed, cryoprotected, cut in 30-µm frontal sections, and immunostained as described previously [38]. Self-made rabbit polyclonal anti-Stau2 antibody [39] was diluted 1:200, additionally anti-GluRD2 (rabbit polyclonal, Alomone, 1:200), anti-vGLUT1 (guinea pig polyclonal, Synaptic Systems, 1:500, Göttingen, Germany), calbindin (mouse monoclonal, Abcam, 1:500), and anti-NeuN (chicken polyclonal, Millipore, 1:500, Darmstadt, Germany) were used. To detect the primary antibody, sections were washed three times in PBS and incubated with secondary antibodies (goat anti-rabbit IgG Alexa Flour 488 labeled (Jackson Immunoresearch, Cambridgeshire, UK), goat anti-guinea pig IgG Cy3 labeled (Jackson Immunoresearch), goat anti-chicken IgY Alexa Flour 647 labeled (Life Technologies, Carlsbad, CA, USA), and donkey anti-mouse IgG Alexa Flour 555 labeled (Life Technologies) diluted 1:500 in blocking solution for 2 h at room temperature. Nuclei were stained by 5-min incubation in 4′,6-diamidino-2-phenylindole (DAPI) solution. Slices were washed three times in PBS and mounted in Fluomount (Sigma-Aldrich, Munich, Germany). Pictures were taken by a confocal SP8 microscope (Leica, Wetzlar, Germany).

*Imaging*: Dendritic stretches of cerebellar Purkinje cells were identified using calbindin staining and the Imaris software package (Bitplane, South Windsor, CT, USA). Fiji 1.50g (PMID 26153368) software was used to outline 20 randomly chosen dendritic stretches per group in the WT and Stau2^GT^ mice at 10 or 13 weeks, respectively. Immunofluorescence intensity of GluD2 was measured in images taken at the same gain level by a confocal microscope (Leica, Germany).

*LacZ-staining*: To monitor gene trap vector insertion in murine brains, we detected LacZ expression by determining the encoded β-galactosidase activity in slices of Stau2^GT^ mouse brains according to the manufacturer’s instructions (InvivoGen, San Diego, CA, USA). The procedure was performed as described previously [9].

### 4.4. RT-qPCR

Total mRNA was obtained from brain samples using TRIzol (Ambion, Carlsbad, CA, USA) according to the manufacturer´s protocol. Genomic DNA was depleted using the Mini RNeasy kit (Qiagen). cDNA was synthesized from purified mRNA by reverse transcription using Superscript III reverse transcriptase (Invitrogen, Carlsbad, CA, USA) according to the manufacturer´s instructions. For qPCR cDNA amplification, Hot Start Taq (New England Biolabs, Ipswich, MA, USA) was used with SYBR Green for amplicon detection. All primers were used with an optimal efficiency rate of 2.0 ± 0.05. Runs were performed on a Lightcycler 96 (Roche Bioanalytics, Basel, Switzerland). Primers used in this study were (5′ to 3′): *Cbln1:* GCTTTCTCTGCCATCAGG and TCTGAGTCAAAGTTGTTCCC, *Grid2:* TGACACCATGAGGATAGAGG and ACCTCACTTATGAAGGATTTGG, *18S*: GAAACTGCGAATGGCTCATTAAA and CCACAGTTATCCAAGTAGGAGAGGA.

### 4.5. Expression Analysis

*Microarray analysis*: RNA was isolated as described above. The samples were processed according to the manufacturer’s instructions (Affymetrix, Santa Clara, CA, USA) and hybridized on a Mouse Gene 2.0 ST Array. Signal intensities were extracted and normalized using RMA (R/bioconductor package ‘oligo’). Samples with log2-expression levels of >5 in at least 3 samples were subjected to differential expression analysis using limma and multiple testing correction according to Benjamini and Hochberg (R/bioconductor package ‘limma’). Data were deposited in the Gene Expression Omnibus (GEO) database (accession no. GSE126996).

### 4.6. Statistics

Data were first tested for Gaussian distribution using the KS-test. Normally distributed data were analyzed by Student’s t-test or one-way ANOVA followed by Bonferroni’s post-hoc test for multiple comparison. Data are presented as mean + SEM. Statistical analysis was performed using the software GraphPad Prism (Version 5, GraphPad, San Diego, CA, USA). *p* < 0.05 (*) was considered statistically significant if not stated otherwise.

## Abbreviations

Cbln1 GluD2 KO LTD RBP Stau2 Stau2^GT^ StG StM StP vGLUT1 WT	Cerebellin1 Glutamate receptor ionotropic delta subunit 2 Knock out Long-term depression RNA-binding protein Staufen2 Staufen2 gene trap Stratum granulosum Stratum moleculare Stratum pyramidale Vesicular glutamate transporter 1 Wild type

## References

[1] Jung H., Gkogkas C.G., Sonenberg N., Holt C.E. (2014). Remote control of gene function by local translation. Cell.

[2] Doyle M., Kiebler M.A. (2011). Mechanisms of dendritic mRNA transport and its role in synaptic tagging. EMBO J..

[3] Kiebler M.A., Bassell G.J. (2006). Neuronal RNA Granules: Movers and Makers. Neuron.

[4] Jarero-Basulto J.J., Gasca-Martinez Y., Rivera-Cervantes M., Ureña-Guerrero M., Feria-Valesco A.I., Beas-Zarate C. (2018). Interactions Between Epilepsy and Plasticity. Pharmaceuticals.

[5] Liu-Yesucevitz L., Bassell G.J., Gitler A.D., Hart A.C., Klann E., Richter J.D., Warren S.T., Wolozin B. (2011). Local RNA Translation at the Synapse and in Disease. J. Neurosci..

[6] Swanger S.A., Bassell G.J. (2013). Dendritic protein synthesis in the normal and diseased brain. Neuroscience.

[7] Hanson K.A., Kim S.H., Tibbetts R.S. (2012). RNA-binding proteins in neurodegenerative disease: TDP-43 and beyond. Wiley Interdiscip. Rev. RNA.

[8] Heraud-Farlow J.E., Kiebler M.A. (2014). The multifunctional Staufen proteins: Conserved roles from neurogenesis to synaptic plasticity. Trends Neurosci..

[9] Popper B., Demleitner A., Bolivar V.J., Kusek G., Snyder-Keller A., Schieweck R., Temple S., Kiebler M.A. (2018). Staufen2 deficiency leads to impaired response to novelty in mice. Neurobiol. Learn. Mem..

[10] Berger S.M., Fernández-Lamo I., Schönig K., Fernández Moya S.M., Ehses J., Schieweck R., Clementi S., Enkel T., Grothe S., von Bohlen und Halbach O. (2017). Forebrain-specific, conditional silencing of Staufen2 alters synaptic plasticity, learning, and memory in rats. Genome Biol..

[11] Hirano T. (2012). Glutamate-receptor-like molecule GluRδ2 involved in synapse formation at parallel fiber-Purkinje neuron synapses. Cerebellum.

[12] Matsuda K., Miura E., Miyazaki T., Kakegawa W., Emi K., Narumi S., Fukazawa Y., Ito-lshida A., Kondo T., Shigemoto R. (2010). Cbln1 is a ligand for an orphan glutamate receptor δ2, a bidirectional synapse organizer. Science..

[13] Mishina M., Uemura T., Yasumura M., Yoshida T. (2012). Molecular mechanism of parallel fiber-Purkinje cell synapse formation. Front. Neural Circuits.

[14] Cheng S., Seven A.B., Wang J., Skiniotis G., Özkan E. (2016). Conformational Plasticity in the Transsynaptic Neurexin-Cerebellin-Glutamate Receptor Adhesion Complex. Structure.

[15] Hashizume M., Miyazaki T., Sakimura K., Watanabe M., Kitamura K., Kano M. (2013). Disruption of cerebellar microzonal organization in GluD2 (GluRδ2) knockout mouse. Front. Neural Circuits.

[16] Miyoshi Y., Yoshioka Y., Suzuki K., Miyazaki T., Koura M., Saigoh K., Kajimura N., Monobe Y., Kusunoki S., Matsuda J. (2014). A new mouse allele of glutamate receptor delta 2 with cerebellar atrophy and progressive ataxia. PLoS ONE.

[17] Coutelier M., Burglen L., Mundwiller E., Abada-Bendib M., Rodriguez D., Chantot-Bastaraud S., Rougeot C., Cournelle M.A., Milh M., Toutain A. (2015). GRID2 mutations span from congenital to mild adult-onset cerebellar ataxia. Neurology.

[18] Miura E., Matsuda K., Morgan J.I., Yuzaki M., Watanabe M. (2009). Cbln1 accumulates and colocalizes with Cbln3 and GluRδ2 at parallel fiber-Purkinje cell synapses in the mouse cerebellum. Eur. J. Neurosci..

[19] Walker M., Mason G. (2018). A comparison of two types of running wheel in terms of mouse preference, health, and welfare. Physiol. Behav..

[20] Brooks S.P., Trueman R.C., Dunnett S.B. (2012). Assessment of Motor Coordination and Balance in Mice Using the Rotarod, Elevated Bridge, and Footprint Tests. Curr. Protoc. Mouse Biol..

[21] Aguiar A.S., Castro A.A., Moreira E.L., Glaser V., Santos A.R.S., Tasca C.I., Latini A., Prediger R.D.S. (2011). Short bouts of mild-intensity physical exercise improve spatial learning and memory in aging rats: Involvement of hippocampal plasticity via AKT, CREB and BDNF signaling. Mech. Ageing Dev..

[22] Fremeau R.T., Troyer M.D., Pahner I., Nygaard G.O., Tran C.H., Reimer R.J., Bellocchio E.E., Fortin D., Storm-Mathisen J., Edwards R.H. (2001). The expression of vesicular glutamate transporters defines two classes of excitatory synapse. Neuron.

[23] Goetze B., Tuebing F., Xie Y., Dorostkar M.M., Thomas S., Pehl U., Boehm S., Macchi P., Kiebler M.A. (2006). The brain-specific double-stranded RNA-binding protein Staufen2 is required for dendritic spine morphogenesis. J. Cell Biol..

[24] Sharangdhar T., Sugimoto Y., Heraud-Farlow J., Fernández-Moya S.M., Ehses J., Ruiz de los Mozos I., Ule J., Kiebler M.A. (2017). A retained intron in the 3′-UTR of *Calm3* mRNA mediates its Staufen2- and activity-dependent localization to neuronal dendrites. EMBO Rep..

[25] Uemura T., Lee S.J., Yasumura M., Takeuchi T., Yoshida T., Ra M., Taguchi R., Sakimura K., Mishina M. (2010). Trans-synaptic interaction of GluRδ2 and neurexin through Cbln1 mediates synapse formation in the cerebellum. Cell.

[26] Zanjani H.S., Vogel M.W., Mariani J. (2016). Deletion of the GluRδ2 Receptor in the Hotfoot Mouse Mutant Causes Granule Cell Loss, Delayed Purkinje Cell Death, and Reductions in Purkinje Cell Dendritic Tree Area. Cerebellum.

[27] Kashiwabuchi N., Ikeda K., Araki K., Hirano T., Shibuki K., Takayama C., Inoue Y., Kutsuwada T., Yagi T., Kang Y. (1995). Impairment of motor coordination, Purkinje cell synapse formation, and cerebellar long-term depression in GluR delta 2 mutant mice. Cell.

[28] Kakegawa W., Miyoshi Y., Hamase K., Matsuda S., Matsuda K., Kohda K., Emi K., Motohashi J., Konno R., Zaitsu K. (2011). D-Serine regulates cerebellar LTD and motor coordination through the δ2 glutamate receptor. Nat. Neurosci..

[29] Hirai H., Launey T., Mikawa S., Torashima T., Yanagihara D., Kasaura T., Miyamoto A., Yuzaki M. (2003). New role of delta2-glutamate receptors in AMPA receptor trafficking and cerebellar function. Nat. Neurosci..

[30] Uemura T., Mori H., Mishina M. Direct interaction of GluRdelta2 with Shank scaffold proteins in cerebellar Purkinje cells. Mol. Cell. Neurosci..

[31] Lebeau G., Miller L.C., Tartas M., McAdam R., Laplante I., Badeaux F., DesGroseillers L., Sossin W.S., Lacaille J.C. (2011). Staufen 2 regulates mGluR long-term depression and Map1b mRNA distribution in hippocampal neurons. Learn. Mem..

[32] Hirai H., Pang Z., Bao D., Miyazaki T., Li L., Miura E., Parris J., Rong Y., Watanabe M., Yuzaki M. (2005). Cbln1 is essential for synaptic integrity and plasticity in the cerebellum. Nat. Neurosci..

[33] Matsuda K., Yuzaki M. (2012). Cbln1 and the delta2 glutamate receptor—An orphan ligand and an orphan receptor find their partners. Cerebellum.

[34] Otsuka S., Konno K., Abe M., Motohashi J., Kohda K., Sakimura K., Watanabe M., Yuzaki M. (2016). Roles of Cbln1 in Non-Motor Functions of Mice. J. Neurosci..

[35] Takeuchi E., Ito-Ishida A., Yuzaki M., Yanagihara D. (2018). Improvement of cerebellar ataxic gait by injecting Cbln1 into the cerebellum of cbln1-null mice. Sci. Rep..

[36] Hamm R.J., Pike B.R., O’Dell D.M., Lyeth B.G., Jenkins L.W. (1994). The rotarod test: An evaluation of its effectiveness in assessing motor deficits following traumatic brain injury. J. Neurotrauma.

[37] Calzolari F., Michel J., Baumgart E.V., Theis F., Götz M., Ninkovic J. (2015). Fast clonal expansion and limited neural stem cell self-renewal in the adult subependymal zone. Nat. Neurosci..

[38] Follwaczny P., Schieweck R., Riedemann T., Demleitner A., Straub T., Klemm A.H., Bilban M., Sutor B., Popper B., Kiebler M.A. (2017). Pumilio2-deficient mice show a predisposition for epilepsy. Dis. Model. Mech..

[39] Heraud-Farlow J.E., Sharangdhar T., Li X., Pfeifer P., Tauber S., Orozco D., Hörmann A., Thomas S., Bakosova A., Farlow A.R. (2013). Staufen2 regulates neuronal target RNAs. Cell Rep..

